# Correction mechanism for Raman spectroscopy in emulsions

**DOI:** 10.1007/s00216-025-05925-w

**Published:** 2025-06-11

**Authors:** Erik Spoor, Matthias Rädle, Jens-Uwe Repke

**Affiliations:** 1 CeMOS Research and Transfer Center, Technical University of Applied Sciences Mannheim, Paul-Wittsack-Str. 10, Mannheim, 68163 Germany; 2 Process Dynamics and Operations Group, Technische Universität Berlin, Straße des 17. Juni 135, Berlin, 10623 Germany

**Keywords:** Disperse phase, Continuous phase, Optical spectroscopy, Raman spectroscopy, UV/VIS spectroscopy, Emulsion measurement

## Abstract

When the concentration of a fluid mixture is measured with Raman spectroscopy in emulsions instead of pure liquids, the signal strength is influenced by the light scattering of the droplets which gives wrong results. This work investigates this influence in the example of a water-toluene-acetone emulsion. For this purpose, the Raman spectroscopy is supported by a scattered light probe, which is intended to quantify the light losses when the dispersed toluene phase increases. The scattered light probe is aligned with the focal point of the Raman probe and detects the light from the 785 nm laser scattered by droplets. The aim is to determine the effects of emulsions on Raman spectroscopy dependent on the concentration of the disperse phase and to determine the acetone concentration of the mixture. The Raman signal decreases with increasing turbidity due to the disperse phase and the concentration of acetone can than no longer be determined from the signal. However, the increase in droplets increases the scattering of the excitation light, whereby a reduction in signal strength is detected. These measurements can be correlated to create a correction function. This makes it possible to correct the measured data of the acetone up to an RMSEP of 1.5 wt%.

**Graphical Abstract:**

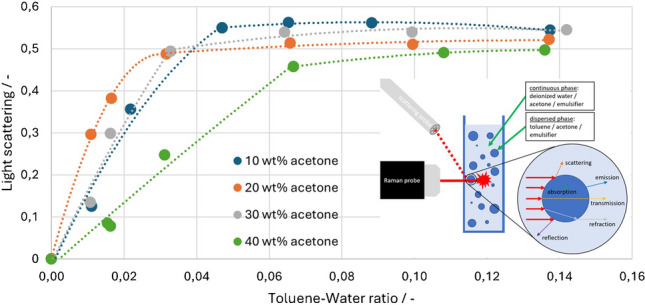

## Introduction

Process analysis technology is a diverse field of measurement methods for analyzing reactants and products in various production plants. Particularly in the analysis of mixtures of substances in the chemical industry, optical measuring systems can provide a detailed insight into the composition of mixtures and monitor the progress of reactions [1–4].

The Raman spectroscopy utilized in this work is molecule-sensitive and provides a spectrum that allows a distinct assignment of peak positions to molecular groups [5, 6]. In addition to this qualitative determination of the composition, the quantity of the individual components can also be determined, as there is a linear relationship between concentration and corresponding peak height [5–7]. The Raman measurement technique utilizes a laser that is focused on a target. Focusing achieves a high energy density at the target location. There, the photons of the laser interact with the molecules of the target, which results in valence electrons being brought to a higher energy level. However, the energy input is not sufficient to reach a stable state, and the energy level drops again causing photons to be emitted from the molecule. These photons are scattered in all spatial directions and have a different frequency, energy, and wavelength compared to the excitation laser. This change in wavelength is also known as the Raman shift and depends on the irradiated molecule group. This Raman shift can be measured using appropriate detectors and creates an output in the form of a spectrum [5, 8–10]. In addition to the ability to differentiate between molecular groups, there are other advantages, such as non-destructive and contactless measurement directly in the process [5, 6, 9, 10]. A disadvantage in the application is the general use of laser modules, which involves increased effort in terms of laser protection. Typical laser powers can be in the range of 500 mW, if particularly high signals are to be obtained. With highly sensitive detectors, however, it is also possible to reduce the laser power to less than 10 mW and, for example, meet explosion protection requirements in accordance with DIN EN 60078-28 [11–13].

For homogeneous and transparent fluids, the use of Raman spectroscopy is already well established. The story is different for disperse and turbid systems. These systems present a particular challenge for optical measurements, as light refraction, scattering or reflection can occur on all droplets and/or particles. On the one hand, this leads to a lower power density of the excitation laser in the focal point, and, on the other hand, the generated Raman signal is deflected on the way to the detector, resulting in a reduced signal yield [14–18]. In order to obtain reliable results, the signal must therefore be corrected. Examples of this are complex calibration systems that take several parameters into account [19, 20], refractive index matching [16, 21], which reduces the effects of boundary layers, or calculating the signals with quantities of substances in the system that remain constant and known during the reaction [14, 22, 23]. Instead of analytical solutions, technical solutions can also be used, such as the use of phase separation cells [24]. However, this leads to more complex process control and time-delayed measurements. All these methods are specific solutions that must be redefined for other chemical systems. The aim of this work is a fundamental analysis of the scattering effects in dispersions and a more general approach to signal correction that can be transferred to new mixtures. For suspensions, the relationship between signal scattering and peak height change has already been discussed using the example of glass beads in ammonium nitrate solutions [25, 26]. Now, the previous findings are to be transferred to an emulsion of water-toluene-acetone, which is already well documented in literature [27–31]. For this purpose, the emulsion is measured using a Raman probe, and the scattered light is quantified using a separate glass fiber probe.

## Material and methods

For the measurement setup (Fig. 1), a MultiSpec Desk ETH with an LS-LD laser cassette (785 nm excitation wavelength) and an SC-CCD RAMAN spectrometer cassette (detection range 319–3213 cm^−1^ with steps of 1 cm^−1^) from tec5 (Steinbach, Germany) were used. The laser can be set to a power of 50 to 500 mW and was regulated to 200 mW for the measurements. The laser is coupled into an RPS785/16-5 probe from InPhotonics (Norwood, MA, USA). The probe has a focal length of 7.5 mm, a spot size of 158 µm, and a numerical aperture of 0.27. The probe is fixed in a rail so that the focal point is 2 mm inside the cuvette from Hellma (Müllheim, Germany). Previous studies have shown that a deeper penetration depth leads to considerable signal loss with higher concentrated dispersions [25]. The backscattered Raman signal is again received by the InPhotonics probe and forwarded to the tec5 spectrometer. A second self-made probe is positioned at an angle above the Raman probe and aligned with the focal point of the Raman probe. This probe consists of a metal casing in which a polished glass fiber bundle is glued. It receives the scattered light at 785 nm and transmits it to an MCS 601 UV-NIR C spectrometer (detection range 190–1015 nm with steps of 0.5 nm and spectral resolution of 2.4 nm) from Zeiss (Oberkochen, Germany). The emulsion data was measured ten times with an integration time of 5 s (Raman) and 30 ms (scattered light) and an average value was calculated from the measurement points. These measurement parameters and deviating parameters from the preliminary measurements are listed in the figure captions.

**Fig. 1:**
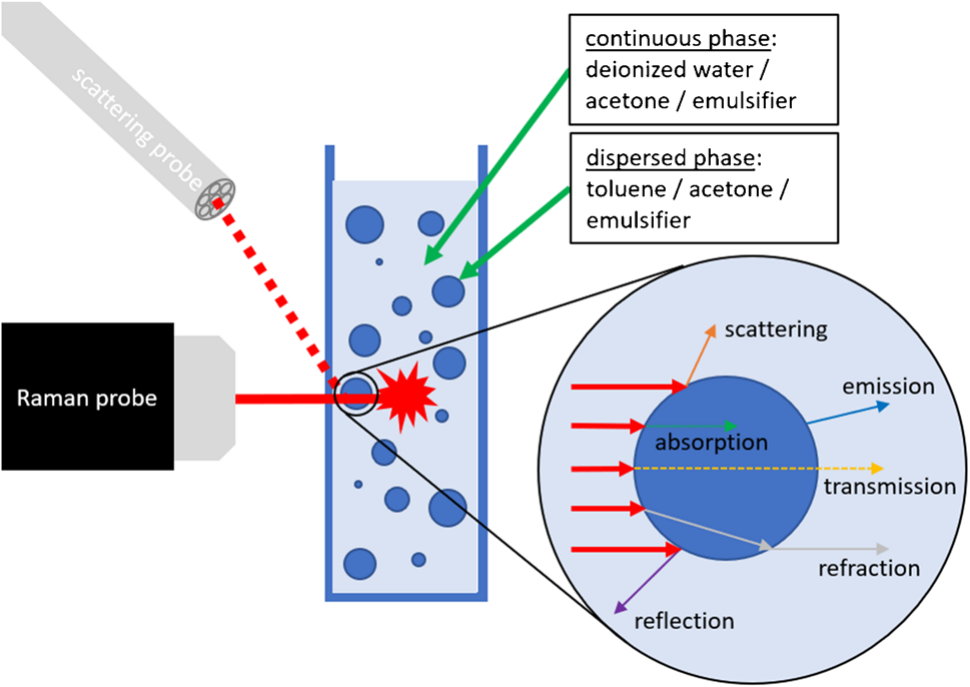
Scheme of the measurement setup with Raman probe, scattering probe, cuvette and examples of light-droplet interaction

The investigated emulsion consists of deionized water, toluene, acetone, and the emulsifier polysorbate20. Water and toluene are not soluble, which means that the toluene component with a lower proportion is present as droplets in the mixture. The ratio of toluene to water varies from 0 to 0.14. To ensure that the emulsion remains stable for the measurements and does not separate, 2 wt% emulsifier (Polysorbate20) is added. In addition, acetone is added in concentrations of 10, 20, 30, and 40 wt%, which is soluble in all other components. Acetone is also the component to be measured, and the peak change is investigated in more detail as a function of the toluene concentration. To ensure that all components are mixed and dispersed with each other, a T 25 disperser from IKA-Labortechnik (Staufen im Breisgau, Germany) is used to emulsify at 8000 rpm for 10 minutes. Figure 2 shows a concentration sequence of 10 wt% acetone, 2 wt% emulsifier, 0–10.56 wt% toluene, and 88–77.44 wt% water. At low toluene concentrations the turbidity increases, and above 3 wt%, no difference in turbidity is visible to the naked eye.

**Fig. 2:**
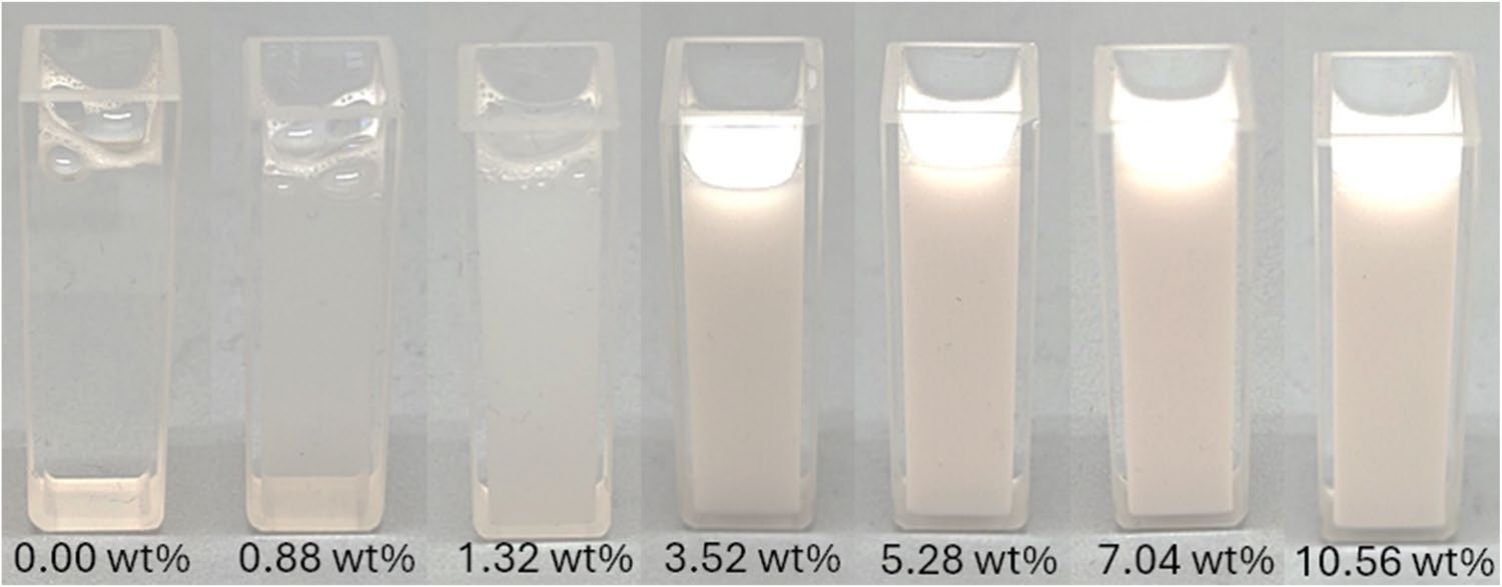
Turbidity of the emulsion with an increasing toluene concentration and a constant acetone concentration of 10 wt%

Figure 3 shows images taken with a Keyence (Mannheim, Germany) VHX-7000 microscope and VH500R zoom objective at 3000× magnification. At low toluene concentrations, even a few droplets are visible, corresponding to the perception that the liquid is still transparent to the naked eye. At concentrations of up to 10 wt% toluene, the number of droplets increases, and the mixture becomes turbid. The size of the droplets is in the range of 1 µm, with individual droplets up to approx. 3 µm.

**Fig. 3:**
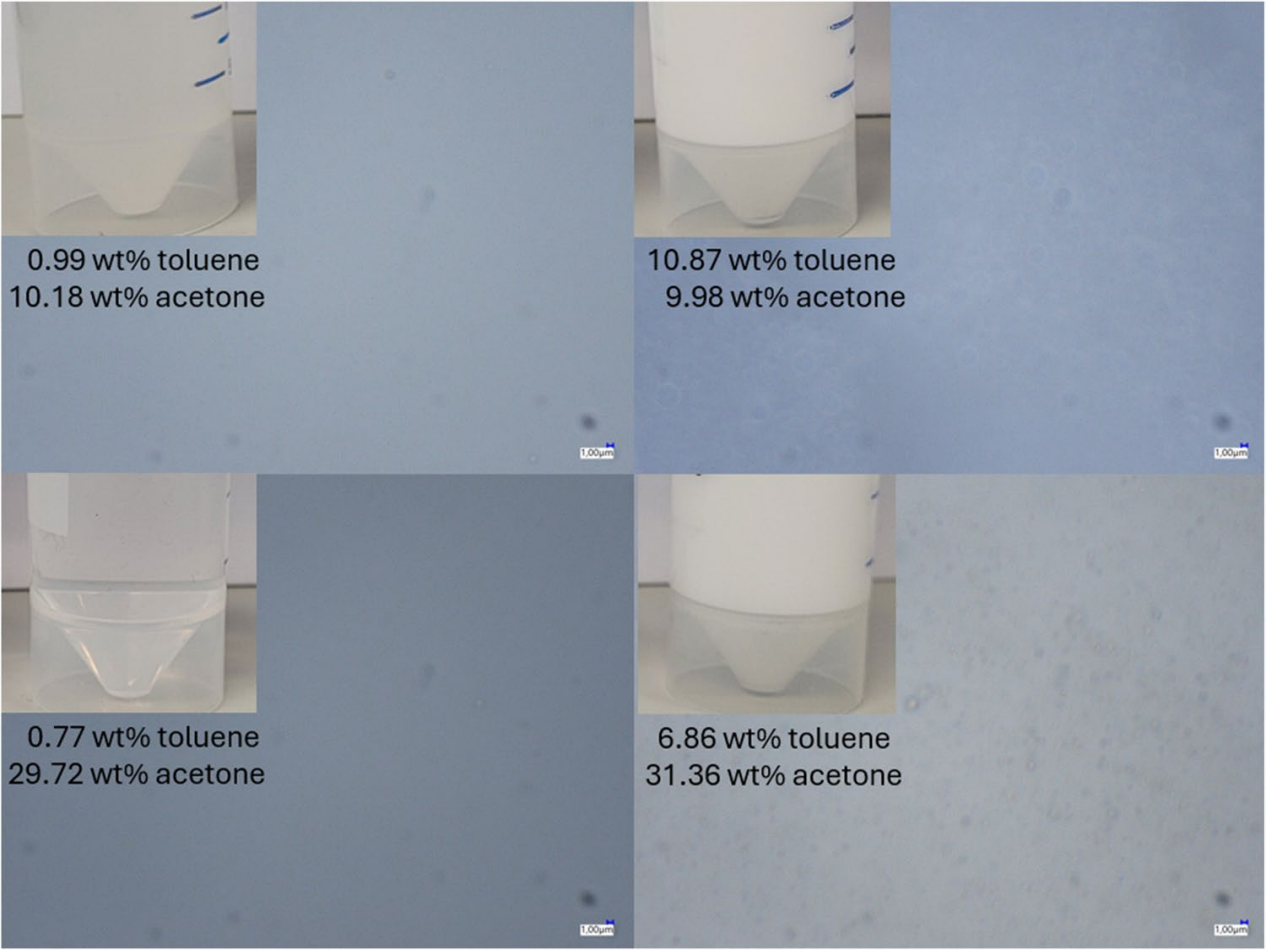
Microscope images of selected emulsions at 10 and 30 wt% acetone, with a Keyence (Mannheim, Germany) VHX-7000 microscope and VH500R zoom objective at 3000× magnification

## Results

### Analysis of the individual components

At the beginning, the individual components of the emulsion are examined to find a relevant spectral range for the analysis (Fig. 4). Acetone (C_3_H_6_O) has its largest peak at 792 cm^−1^, which is due to the C-C stretching. In addition, the CH3 compounds result in peaks at 1442 and 2930 cm^−1^ and the C=O bond results in a peak at 1715 cm^−1^ [32]. The most characteristic peaks of toluene (C_7_H_8_) are mainly due to the benzene ring of the compound, which produces a signal at 790 and 1008 cm^−1^ [27]. The characteristic peak of water (H_2_O) or the OH band is at approx. 3400 cm^−1^ and thus just outside the measurement range [33, 34]. In all other ranges, the signal is low enough in proportion to toluene and acetone to have no significant cross-influence. Polysorbate20 (C_58_H_114_O_26_) shows distinct peaks in the range from 791 to 1008 cm^−1^, which overlap with the peaks of the toluene signal. However, due to the low intensity of the signals and the constant concentration in all samples, it can be concluded that this component is not a significant interfering factor.

**Fig. 4:**
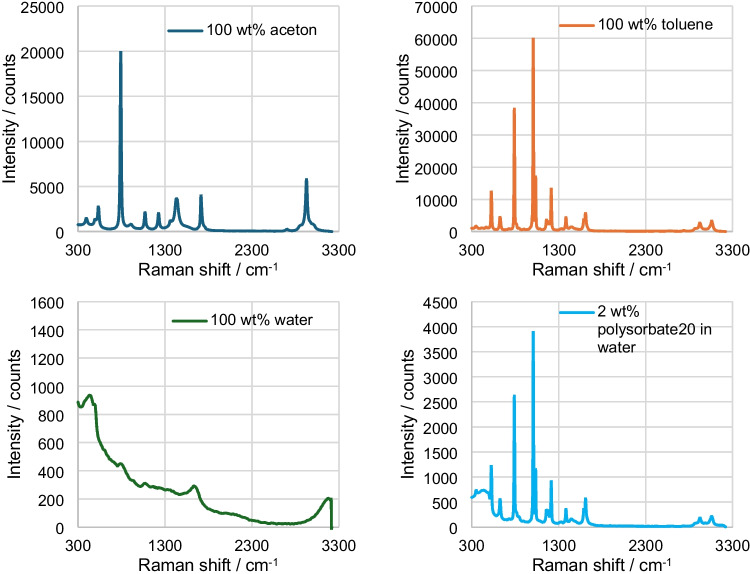
Raman spectrum from 300 to 3300 cm^−1^ of acetone, toluene, water, and 2 wt% polysorbate20 in water; integration time: 1 s, average: 3

The aim is to determine the acetone concentration with increasing toluene content. To be able to do this independently of all other components, a range must be evaluated that has only one acetone peak. The only suitable range here is the characteristic peak at 1715 cm^−1^, as shown in more detail in Fig. 5.

**Fig. 5:**
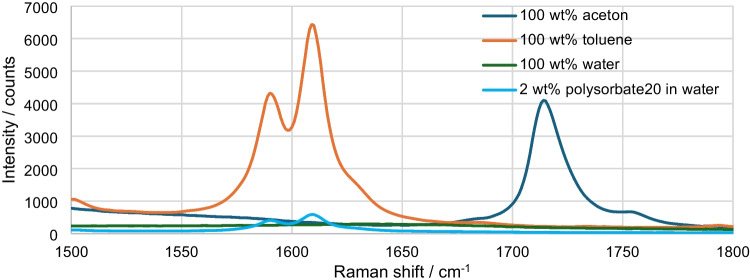
Raman spectrum from 1500 to 1800 cm^−1^ of acetone, toluene, water, and 2 wt% polysorbate20 in water; integration time: 1 s, average: 3

After analyzing the individual components, a calibration is performed for the acetone concentration. Since the acetone is distributed between the water and the toluene phase, the two phases are measured separately as homogeneous mixtures. Figure 6a shows a series of mixtures from 0 to 25 wt% acetone in water (with 2 wt% polysorbate20) and Fig. 6b shows a series of mixtures from 0 to 25 wt% acetone in toluene (with 2 wt% polysorbate20). The peak maximum at 25 wt% acetone is approx. 5000 counts in both series and is reduced to 1394 counts in water at a concentration of approx. 6.71 wt% acetone and to 1169 counts in toluene at approx. 5.91 wt% acetone. It is noticeable that there is a shift in the peak position, with the peak of acetone in water at 1703 cm^−1^ and in toluene at 1720 cm^−1^.

**Fig. 6:**
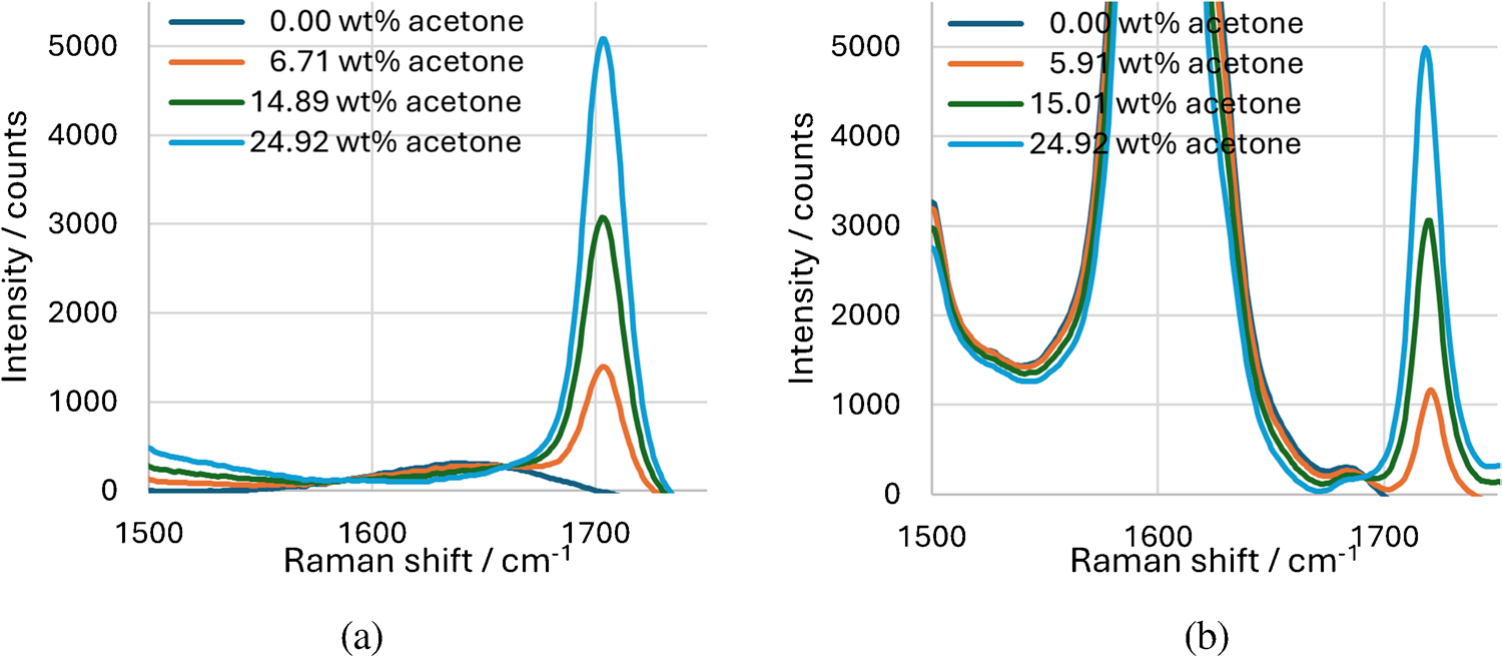
Raman spectrum of **a** acetone in water and **b** acetone in toluene; integration time: 5 s, average: 10

In order to minimize measurement inaccuracies due to offset changes, the maximum of the acetone peaks (${I}_{max}$) is baseline corrected. For this purpose, the mean value of the peak base at 1670 (${I}_{{1670cm}^{-1}}$) and 1760 cm^−1^ (${I}_{{1760cm}^{-1}}$) is subtracted from the value of the peak maximum at 1703 and 1720 cm^−1^ in Eq. 1.

1 $${I}_{acetone}={I}_{max}-\frac{{I}_{{1670cm}^{-1}}+{I}_{{1760cm}^{-1}}}{2}$$

Finally, a regression can be established from the acetone signal (${I}_{acetone}$) to later calculate corrected Raman signals back into concentrations. To do this, the acetone concentrations (ω_acetone_) are plotted against the Raman intensity in Fig. 7 and a mean regression is formed from both concentration series.

**Fig. 7:**
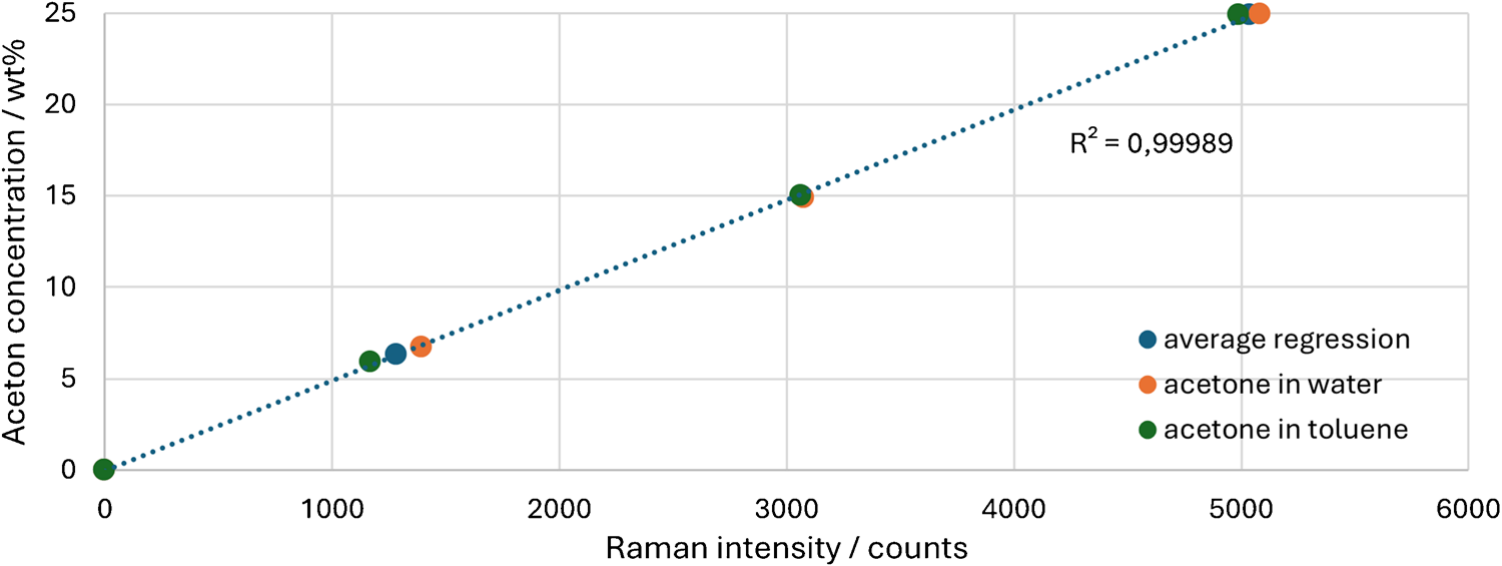
Calibration of the acetone peak in water and toluene with regression

Figure 7 results in regression Eq. 2, which can later be used to convert the corrected Raman intensities (${I}_{acetone-corr}$) into an acetone concentration:

2 $${\omega }_{acetone}=0.00494\cdot {I}_{acetone-corr}+0.04501$$

### Evaluation of the disperse system

All Raman data were acquired with a laser power of 200 mW and an integration time of 5 s. The scattered light data was acquired every second with an integration time of 30 ms and averaged over 10 values to generate a single value. Each measurement points of the Raman and scattered light measurements was additionally averaged from 10 individual measurements. From each of these ten measuring points, a standard deviation can be determined for the signal of approx. 15.5 counts (1.77%) for the Raman measurement and 181.8 counts (0.58%) for the scattered light measurement.

The results of the Raman measurements are shown in Fig. 8. The average standard deviation of the measurements is 1.64%. As expected, the data without the disperse phase or without toluene in the mixture are in line with the linear trend line in Fig. 7. As the toluene concentration increases, the proportion of the disperse phase in the overall mixture increases and light is scattered more significantly, causing the measurement signal to drop. This basic process can be seen in all four series of measurements. However, the measurement series with 40 wt% acetone deviates, as the first measurement points decrease significantly less. This is due to the fact that with a proportion of 40 wt% acetone, the turbidity that can be seen with the naked eye only begins with a higher proportion of toluene. While with 10 to 30 wt% acetone a slight turbidity is already visible at 0.88 wt% toluene, with 40 wt% acetone a comparable turbidity is only visible above 1 wt% toluene. This results from the fact that acetone dissolves in both phases and thus the refractive index difference between the phases decreases. For the series of measurements from 10 to 30 wt% toluene, a minimum is reached at approx. 1 wt% toluene, which lies at approx. 293.6 counts (10 wt% acetone) and 716.4 counts (30 wt% acetone). The measured values then rise again slightly with increasing toluene concentration to 390.2 counts (10 wt% acetone) and 907.3 counts (30 wt% acetone). For 40 wt% acetone, this minimum is only reached at approx. 3.6 wt% toluene. The explanation for the lack of further decrease in the signal is that a state of maximum uniform scattering of light in all spatial directions is reached. A slight increase in the signal may be due to the fact that the disperse phase generates a clearer measurement signal as the proportion increases, without more signal being lost due to scattering. However, as this effect primarily relates to the toluene concentration and the acetone concentration remains constant, this only results in an increase of a few hundred counts and has no significantly greater influence on higher concentrations than shown.

**Fig. 8:**
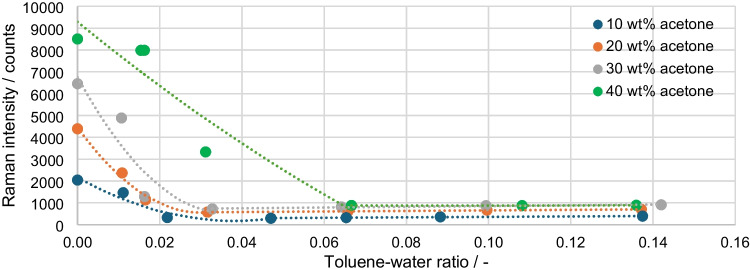
Raman intensity of the acetone peak over the toluene-water ratio/integration time: 5 s, average: 10

The scattered light measurements, which were carried out at the same time as the Raman measurements, are calculated according to Eq. 3, analogous to the previous paper, which examined suspensions in the same way [26]. Equation 3 is based on the Beer-Lambert law [6], which for the purpose of the application, uses the reciprocal of the fraction in the logarithm. The Beer-Lambert law normally calculates extinction, which must increase for the Raman signal, as the measured data decreases with increasing disperse phase. However, the scattered light probe measures the signal lost for the Raman measurement, whereby an increase in scattered light is detected. If the Beer-Lambert law was applied correctly, a decrease in extinction would be calculated. For this reason, the formula was adapted to reflect the perspective of the Raman probe and to calculate an increasing scattered light signal.

3 $${E}_{\lambda }={\text{log}}_{10}\left(\frac{I}{{I}_{0wt\%\;toluene}}\right)$$

The calculated values of the light scattering are plotted in Fig. 9 and show a pattern that is opposite to that of the Raman signals. The average standard deviation of the measurements is 0.7%. The effects occurring here are the same as already explained. As the disperse phase increases, the Raman signal decreases and the corresponding scattered light signal increases. The later and flatter rise of the 40 wt% acetone curve can also be observed.

**Fig. 9:**
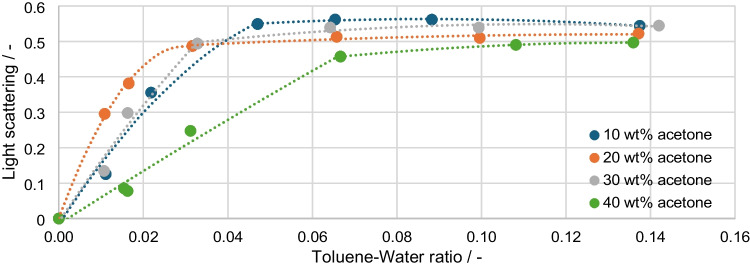
Calculated light scattering over the toluene-water ratio/integration time: 30 ms, average: 10

The Raman values are calculated as the change in peak height relative to the value without disperse phase and plotted against the light scattering to obtain a correction factor as a function of the measured scattered light signal. Figure 10a shows all four curves and their regressions. The theoretical expectation is that there is a correlation between signal loss and light scattering that is independent of the acetone concentrations. For the acetone concentrations of 10, 30, and 40 wt%, this can be confirmed to a first approximation. The 20 wt% series of measurements represents a deviation that also produces the same results in repeated measurements. For all measurement series, the increase in the Raman signal and the reduction in the peak height change at light scattering levels above 0.49 is more apparent in this plot. The 30 wt% acetone measurement series shows the strongest change, with a maximum of 9.01 and a drop of 1.9. For the most accurate calibration, two calibration ranges (Fig. 10b) are defined, which are separated at a scattering of 0.49. Four representative measuring points are selected for each calibration range, and the trend line is output as a correction function.

**Fig. 10:**
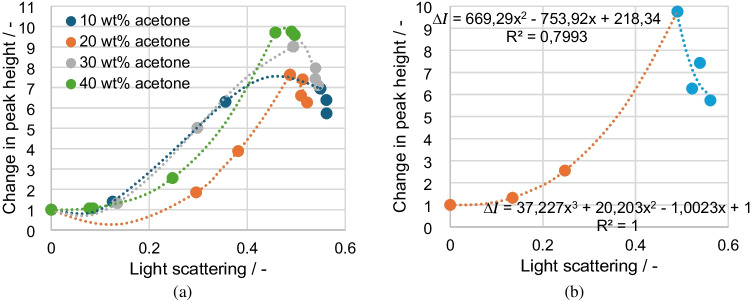
Change in peak height plotted over the light scattering for **a** all measurement points and **b** selected measurement points for calibration purposes

From the correction functions in Fig. 10b, the change coefficient ∆*I* can be measured as a function of the measured light scattering and finally the corrected acetone concentration ${I}_{acetone-corr}$ can be calculated using Formula 4.

4 $${I}_{acetone-corr}={I}_{acetone}\cdot \Delta I$$

The corrected acetone concentrations are shown in Fig. 11. Theoretically, four horizontal lines should form at the four acetone concentrations of 10, 20, 30, and 40 wt%. For the concentrations 10, 30, and 40 wt%, this is indeed true to a rough approximation. However, the 20 wt% deviates significantly and results in consistently higher concentrations around 25 wt% and for a ratio of 0.11, even around 40 wt%. When looking at Fig. 10, this deviation is to be expected, as the 20 wt% measuring points lie below the correction line and are therefore overcorrected. Compared with the fact that, without correction, the Raman values would indicate a concentration of 3 wt%, the correction can still be regarded as an approximate value despite the deviation. The generally largest deviations of the prediction are in the range of the toluene-water ratio of 0.10 to 0.22. The measured value at 30 wt% acetone and a ratio of 0.016, for example, is 8 wt% below the target value.

**Fig. 11:**
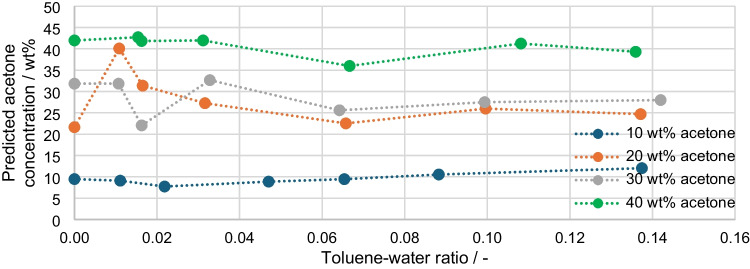
Predicted acetone concentration plotted over the toluene-water ratio

To determine the general quality of the prediction, the root mean square error of the prediction (RMSEP) is calculated for the individual measurement points and the total data sets according to Formula 5 [35].

5 $$RMSEP= \sqrt{\frac{1}{N}\cdot \sum_{i=1}^{N}{({y}_{i}-{\overline{y} }_{1})}^{2}}$$

Figure 12 shows the predicted acetone concentration (a) compared to the corresponding RMSEP values (b). The bars for a toluene-water ratio of 0.00 represent a quasi-ideal result, but it is to be expected that the measurements with the disperse phase show a higher inaccuracy due to the scattering effects, which can vary due to different droplet size distributions. As can also be seen from Fig. 11, the largest RMSEP is at 0.01 and 20 wt% acetone. For a ratio of 0.02, there are also noticeably high deviations above 9 wt% for 20 and 30 wt% acetone. All other measured values show RMSEP of less than 6 wt% and more than half of the measured values are even below 2 wt%.

**Fig. 12:**
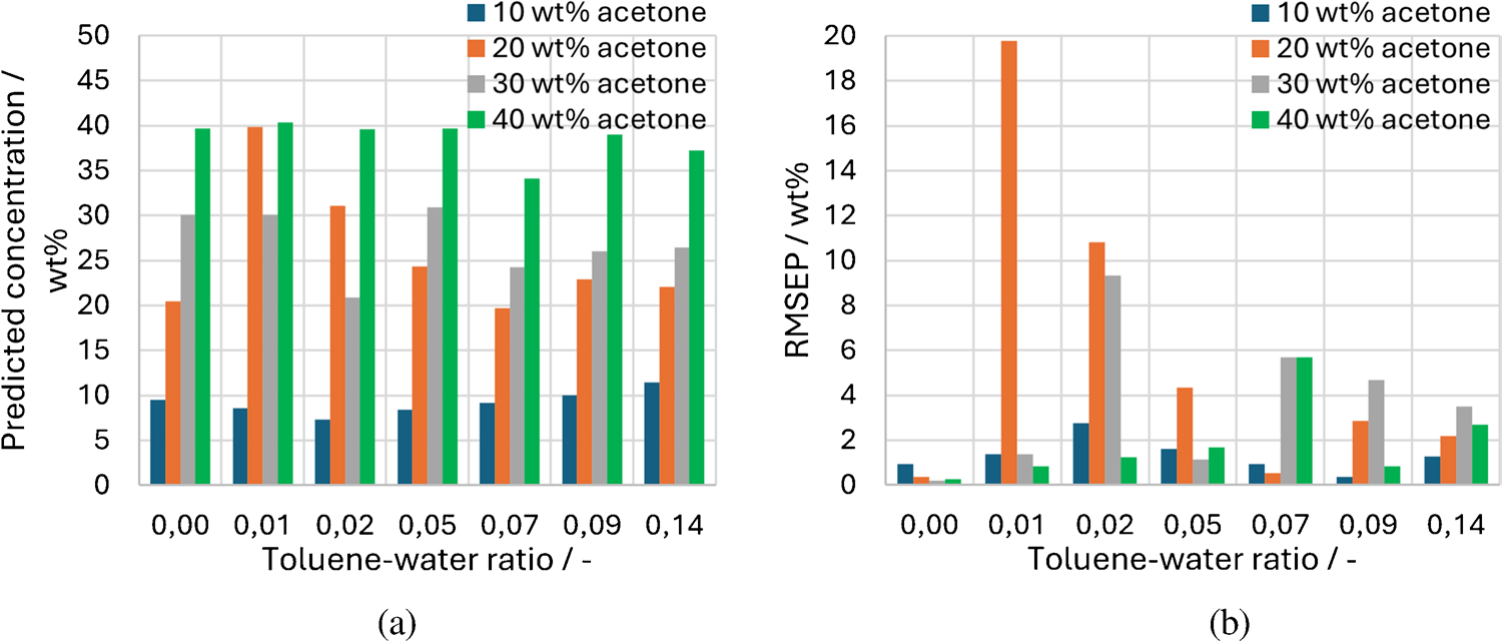
**a** Predicted acetone concentration plotted over the toluene-water ratio with **b** Corresponding RMSEP values

The final overview of all RMSEP is shown in Table 1 for each acetone series and in total. As already explained, the 20 wt% measurement series shows the largest RMSEP with a total of 8.78 wt%, which is significantly influenced by two of the seven measurement points. The 10 wt% acetone series shows the best RMSEP with 1.51 wt%.

**Table 1:** Overview of the RMSEP of the predicted acetone concentrations

Acetone concentration (wt%)	RMSEP (wt%)	Overall RMSEP (wt%)
10	1.51	5.21
20	8.78	
30	4.73	
40	2.54	

## Discussion

The aim of the paper was to investigate the Raman signal in emulsions with an increasing content of the disperse phase. For this purpose, measurement series of water-toluene-acetone-surfactants were mixed, emulsified and measured. In addition to the Raman probe, the signal scattering at 785 nm is also measured with a scattered light probe. As expected, the Raman signal decreases with increasing proportion of the disperse phase, and the signal of the scattered light measurement reacts in the opposite way and increases. The combination of both measured values leads to a correction function that predicts the real acetone concentrations with a total RMSEP of 5.21 wt%. The 20 wt% measurement series leads to a significant decrease in the results, as it has an RMSEP of 8.78 wt% when considered separately. The largest deviations in the prediction are also at concentrations below 1.5 wt% toluene. When transferred to real processes, this would, therefore, only involve a small concentration range, which may not be significant. When transferring to a real process, a more specific calibration can also take place, in which work is carried out in narrower, already expected concentration ranges.

Previous work has dealt with comparable investigations, but measured suspensions [25, 26]. This simplified substance system, which used glass beads as the disperse phase, offered the advantage of a constant and known particle size distribution, as well as a disperse phase that does not generate its own spectrum. The emulsion, on the other hand, has a particle size distribution that is dependent on the emulsification time and the emulsifier, for example. The disperse phase also generates its own spectrum, which overlaps with the spectrum of the continuous phase. In addition, the acetone component to be measured dissolves in both phases. All these factors lead to the emulsion posing a greater measurement challenge than the suspension. This is also reflected in the RMSEP, which was 1.95 wt% for the suspension and 5.21 wt% for the emulsion. Peak changes up to a maximum factor of 10 have been measured for both dispersions. After that, the Raman signal formed a plateau and no longer changed significantly. The light scattering of the suspension was 2.5 in this range, whereas it was approx. 0.5 for the emulsion. This clear difference in the scattered light signal, despite the same change in peak height, is possibly due to the scattered light probe. For this publication, a new light scattering probe was used, which was calibrated to a different signal strength during installation. A significant difference between the course of the dispersions is that after the maximum change in peak height, the emulsion signal decreases again, while the Raman signal increases slightly. One explanation for the difference of the suspension and emulsion data is, that this area was not measured in the suspension, as the smallest particle size was 2 µm. The emulsion, on the other hand, has droplet sizes below 2 µm, which may result in altered scattering properties. These effects must be investigated in more detail in the future in order to make more precise statements about the signal curves.

An example of a possible future application of the method could be the hydroformylation of decene [24]. In the past, the fluid was fed from the reactor into a phase separation cell, where the phases were measured individually using a Raman probe. In the future, a process could be established that analyzes the substances directly in the reactor, thus saving time and simplifying process control. Another application could be, for example, polymerization processes in which standards are calibrated using Raman spectroscopy [19]. This standard is often a peak of water, as its content remains constant. However, depending on the measurement series, the water signal can be weak and therefore difficult to evaluate. In addition, this requires a spectrometer which is able to measure a large enough range of the spectrum to record the corresponding data. Calibration via the scattered light, on the other hand, would provide a significantly higher signal that can always be recorded using comparably low-cost photometers.

With this correction method, it is therefore possible to correct Raman signals in an emulsion without using complex algorithms or setups. The results demonstrate the basic principles of the method and can lead to even more precise concentration predictions with more precise calibration to relevant measurement ranges.

## Data Availability

The data presented in this study are available on request from the corresponding author.
